# Effect of a smartphone-based physical intervention on depression, fitness factors and movement characteristics in adults

**DOI:** 10.1186/s12889-024-20088-6

**Published:** 2024-09-27

**Authors:** Hyungsook Kim, David Michael O’Sullivan

**Affiliations:** 1 Department of Data Science, Hanyang University, Seoul, Republic of Korea; 2 HY Digital Healthcare Center, Hanyang University, Seoul, Republic of Korea; 3 Department of Physical Education, Seoul National University, Gwanak-ro, Gwanak-gu, Seoul, 08826 Republic of Korea

**Keywords:** Mobile intervention, Mental health, Physical activity, Grip strength, Exercise

## Abstract

**Background:**

Physical activity has been shown to correlate with mental health and a reduction in symptoms of depression. However, the majority of research has focused only on the effects of either aerobic or nonaerobic exercise on depressive symptoms, while the use of novel technological innovations such as mobile phone-based activity programs and their effects on movement characteristics are underrepresented. This study had two objectives: (1) to investigate how effectively 4 weeks of mobile phone-based physical activity can affect depressive scores (CES-10-D and PHQ) and fitness levels and (2) to investigate the whether 4 weeks of mobile phone-based physical activity affected participants’ movement characteristics.

**Methods:**

A total of 31 participants were included and divided into an exercise group (*n* = 21) and a control group (*n* = 10). The exercise group was instructed to use a mobile phone-based exercise program 5 times per week for 4 weeks. Pre- and post-exercise, the participants’ depression score (CES-10-D, PHQ9), fitness level (YMCA, grip strength) and movement characteristics (postural sway, movement ROM, movement speeds, etc.) for three Azure Kinect physical activity games based on different fitness factors (balance game, cardiovascular game, reaction game) were measured.

**Results:**

Mixed model ANOVA revealed significant differences between pre- and post-intervention depression scores on the PHQ9 (*P* = .001) and CES-10-D (*P* < .001) in both the exercise group and the control group, but not between groups. In terms of movement characteristics, there was an increase in body sway (*P* = .045) and vertical head movement (*P* = .02) in the cardiovascular game jogging condition for the exercise group. In the reaction game, the exercise group showed a significant reduction in the number of mistakes (*P* = .03). There were no other significant differences for the other variables.

**Conclusion:**

The results revealed no differences in the reduction in depression scores between the exercise group and the control group. However, this study showed that a mobile phone-based physical activity intervention affects in-game movement characteristics such as body sway and vertical head movement and therefore may show the potential of using activity-promoting mobile games for improving movement.

## Background

Recent research has shown substantial increases in depression due to the COVID-19 pandemic for a wide range of age groups, including adolescents [1], adults [2] and elderly individuals [3]. This phenomenon has been observed worldwide and has been reported in over 204 countries [4], such as the United States [5], Korea [6, 7], India [8] and Australia [9]. Since the pandemic, a vast number of studies have reported the negative effects of COVID-19-related lockdowns on the physical activity habits of children with asthma [10], children with congenital heart disease [11] and adults, whose anxiety and suicidal thoughts [12] were reported to increase due to a lack of physical activity, changes in appetite and sleep.

The inverse relationship between the amount of physical activity and depression symptoms is well known [13–15], with reduced depression following exercise reported for both children and adults [13]. Physical activity can therefore be identified as one of the main risk factors for the management of mental disorders such as depression [14]. A multitude of papers have reported positive effects of increased physical activity on depressive symptoms [16–20], with meta-analytic evidence suggesting that physical activity has a protective effect [15] and can serve as an effective add‐on treatment to usual care [13]. The positive effects of physical activity occur through multiple changes in the brain, including changes in both biological and psychosocial pathways [17]. Following exercise, cellular downstream processes are stimulated by neurotrophins and can improve brain function in areas associated with depression and stress regulation [17]. These effects make physical activity a useful treatment for depressive disorders that is comparable to the effects of antidepressants [16]. The mechanism of antidepressant treatments and the effect of exercise are believed to overlap, with both having an impact on neurogenesis via similar pathways [17].

Currently, the majority of evidence linking depression and physical activity primarily reports the relationship between symptoms and the amount of physical activity. Since 1984, it has been reported that strenuous aerobic exercise [21] has positive effects on depressive symptoms, with more recent publications showing that the addition of resistance training may provide additional benefits for depression [22, 23]. However, the use of novel technological innovations, such as mobile phone-based activity programs, and their possible benefits for treating depressive disorders are underrepresented in current research and need further investigation. With substantial obstacles, such as lockdowns, forcing people to stay home and close fitness centers, preventing people from participating in aerobic and resistance training, novel technological innovations are being used to make physical activity interventions more accessible and more fun for participants at home [24]. It has been reported that the older population, who self-quarantined during COVID-19, showed a decline in physical activity due to reduced motivation, as well as a lack of space and professional instruction [25]. To counteract this trend, there has been a substantial increase in the number of serious games developed for depression, with one review highlighting a significant effect of reduced depression if a serious game includes physical activity [26]. Despite these results, another study reported the need for high-quality RCTs to reduce the high risk of bias in individual studies and to provide more robust evidence for the use of serious games to alleviate depression [27].

In addition to its benefits on mental health, physical activity is suggested to be included in medical screening as an aid for the identification of individuals at high risk for depressive symptoms [28]. Research shows that depression and poor mood influence human movement patterns, leading to alterations in gait characterized by a reduction in arm swing and walking speed, less vertical head movement, increased body sway and a slumped body posture compared to healthy controls [29–32]. These results indicate that depressive disorders are manifested and observable in specific gait patterns [29, 30], making movement characteristics a possible indicator in the detection of depression. Unfortunately, these studies focused only on the effect of depressive symptoms on gait characteristics and did not investigate how other forms of physical activity and movement are affected by mental health. It also remains unclear how physical interventions affect these altered movement characteristics and whether they are reversible. More research on how depression affects movement other than walking and on the effect of alternative physical interventions, such as serious games, on altered movement kinematics in depressive patients is needed.

To gain a better understanding of the relationship between depressive symptoms and physical activity, this study has both primary and secondary objectives. The first objective was to investigate how effectively 4 weeks of mobile phone-based physical activity can affect depressive scores (CES-10-D and PHQ) and fitness levels. The second objective was to investigate whether 4 weeks of mobile phone-based physical activity affected participants’ movement characteristics.

## Methods

### Study design and participants

A two-group (exercise group vs. control group) pretest-posttest study design was used to investigate the effects of a 4-week mobile phone-based physical activity intervention on depressive symptoms, fitness level and movement characteristics. The analyzed data for this study were extracted from a clinical trial performed at Hanyang University (Seoul, South Korea) to compare the behavioral characteristics of depressed patients with those of nondepressed controls. Participants for the original trial were recruited at Hanyang University via convenient sampling both offline and online through the university’s internet website and by posters throughout the university campus. Only participants over 19 years of age were eligible to participate in this study. The exclusion criteria included participants with a diagnosis of comorbid psychiatric disorders, participants suspected of drug abuse, pregnant women and those who were believed to not be eligible for ethical reasons. Participants were randomly divided into an exercise group and a control group. The exercise group was instructed to use a physical activity promotion program accessible via their smartphone 5 days a week (Monday to Friday, with daily reminders sent by SMS) for a duration of 4 weeks. To compare the effect of the intervention, outcome variables (depression score, fitness level, movement characteristics) were measured twice, once before the 4-week program and once after. Prior to starting the intervention, all participants were introduced to the study procedures both verbally and in writing via Hanyang University Hospital (HYUIRB-202203-010-2) IRB-approved informed consent forms in their native language, Korean. To address the first objective of this study, an assessment of participants’ depression scores and fitness levels was performed before and after the intervention. Depression scores were measured using the CES-10-D and PHQ-9 depression questionnaires [6]. To evaluate participants’ current fitness levels, performance in the YMCA step test and grip strength were recorded. According to the second objective of this study, movement characteristics within three different activity-based serious games were captured by three Azure Kinect cameras and analyzed once before and once after the 4-week intervention.

### Serious game design

Three different serious games were designed by Hanyang University to assess participants’ movement characteristics before and after the 4-week mobile phone intervention. The game design was based on the inclusion of specific movement tasks representing different core fitness factors (balance, cardiovascular endurance, reaction time) that were required to complete the games. The first step of developing the games included the identification of common behavioral characteristics of depressed patients. Relevant characteristics were derived through a review of literature reporting impaired movement kinematics within depressed patients and through consultations with mental health experts, such as psychiatrists and psychologists. In the second step, movement tasks that best represented those characteristics were selected by physical activity and movement specialists. Therefore, a balance task, a cardiovascular endurance task and a reaction task were implemented in the exergames since these motor skills are commonly reported to be impaired in people with depression [32]. Each of the three games took 5 min to complete and consisted of several parts, each differing in the required intensity and difficulty. A detailed description of the different parts is given below in the comprehensive explanation of each game.

**Fig. 1:**
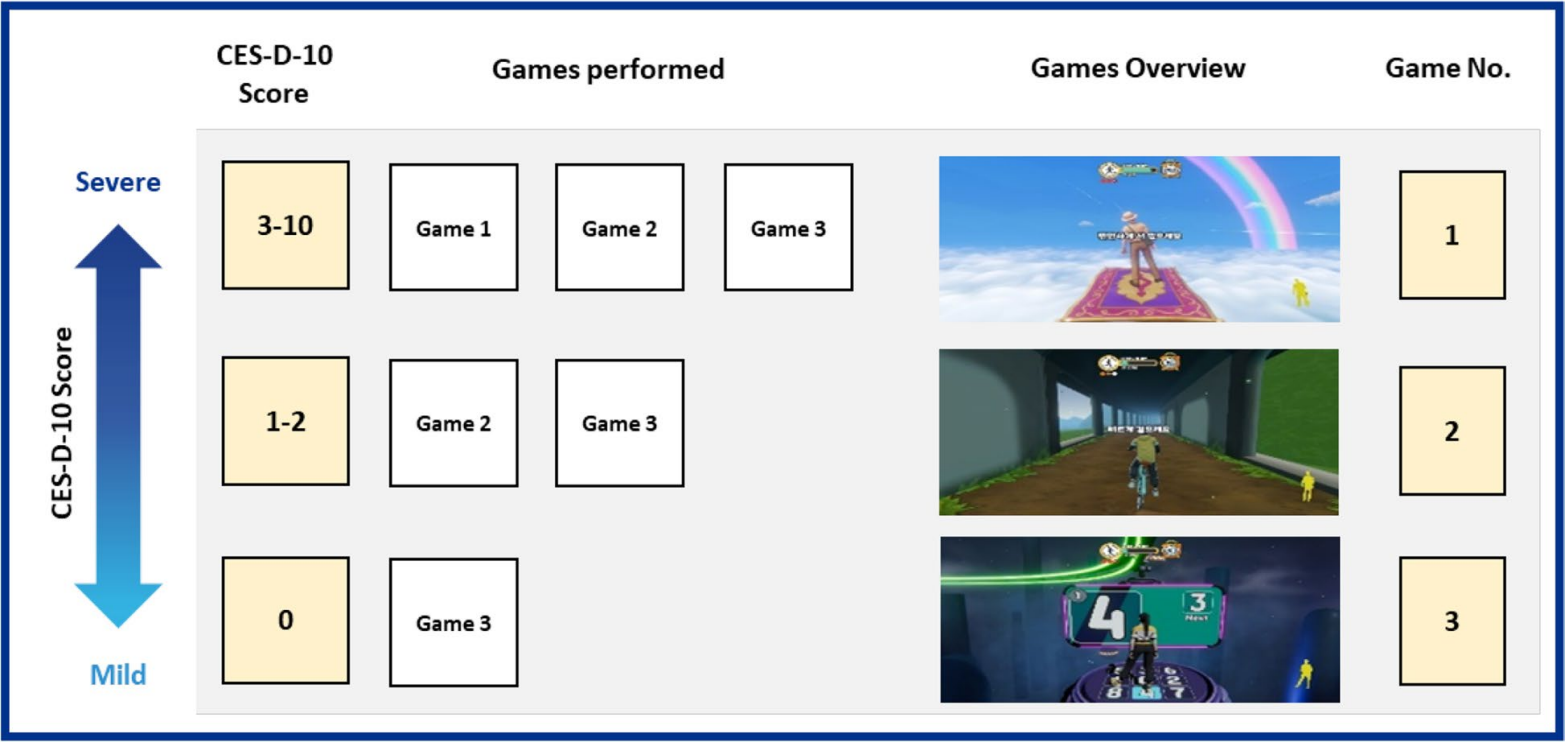
Game selection according to depression score with the number of performed games increasing for individuals with higher CES-D-10 scores

In the initial trial from Hanyang University, the number of games performed varied among participants according to their depression score, with more severe depression scores indicating that they played more games. Participants with a CES-D-10 score greater than or equal to 3 performed all three games, participants with a score of 1–2 performed the first two games, and participants with a score greater than 0 performed only game 3. An overview of game selection according to depression score is given in Fig. 1. For the objectives of this study, we considered participation in all three games essential since each of the games required a motor skill commonly reported to be altered in depressed patients. Thus, only data from participants performing all three games were extracted from the initial data set and included in the analysis, leading to the exclusion of participants with a CES-10-D score smaller than three. After excluding these participants, there were no differences in game selection or game setup among the participants included in this study.

### Game setup

**Fig. 2:**
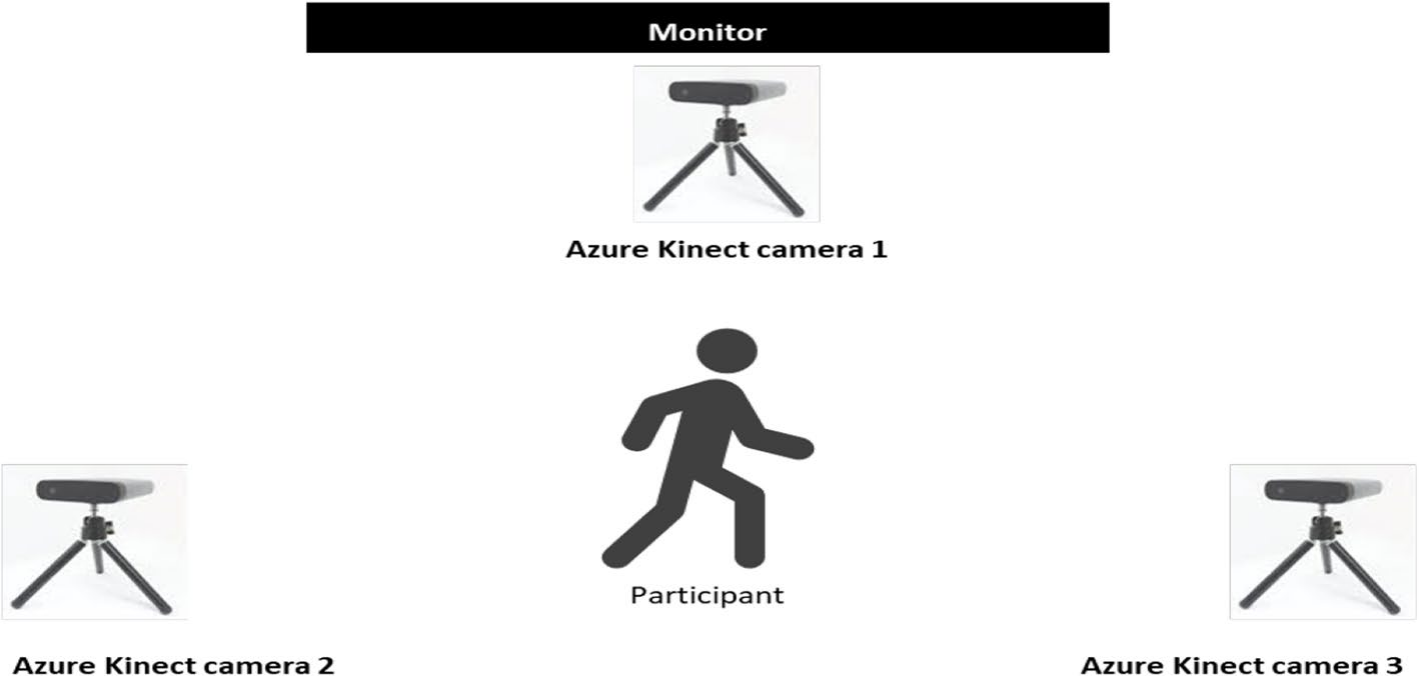
Setup of the monitor and the three Azure Kinect cameras while performing the exergames

The games were performed at Hanyang University both before and after the intervention, and body motion was tracked by three Azure Kinect cameras. Each game was displayed through a flat screen monitor placed in front of the participants. The Azure Kinect cameras were placed in the front (in front of the monitor), to the right and left sides of the participants, allowing motion to be captured from these three perspectives. Despite being surrounded by cameras, the participants were provided with adequate space to move freely without limitations. Although the front camera was placed right in front of the monitor, none of the participants reported limited visibility of the screen while playing the games. The game setup is illustrated in Fig. 2.

### Game explanation

**Fig. 3:**
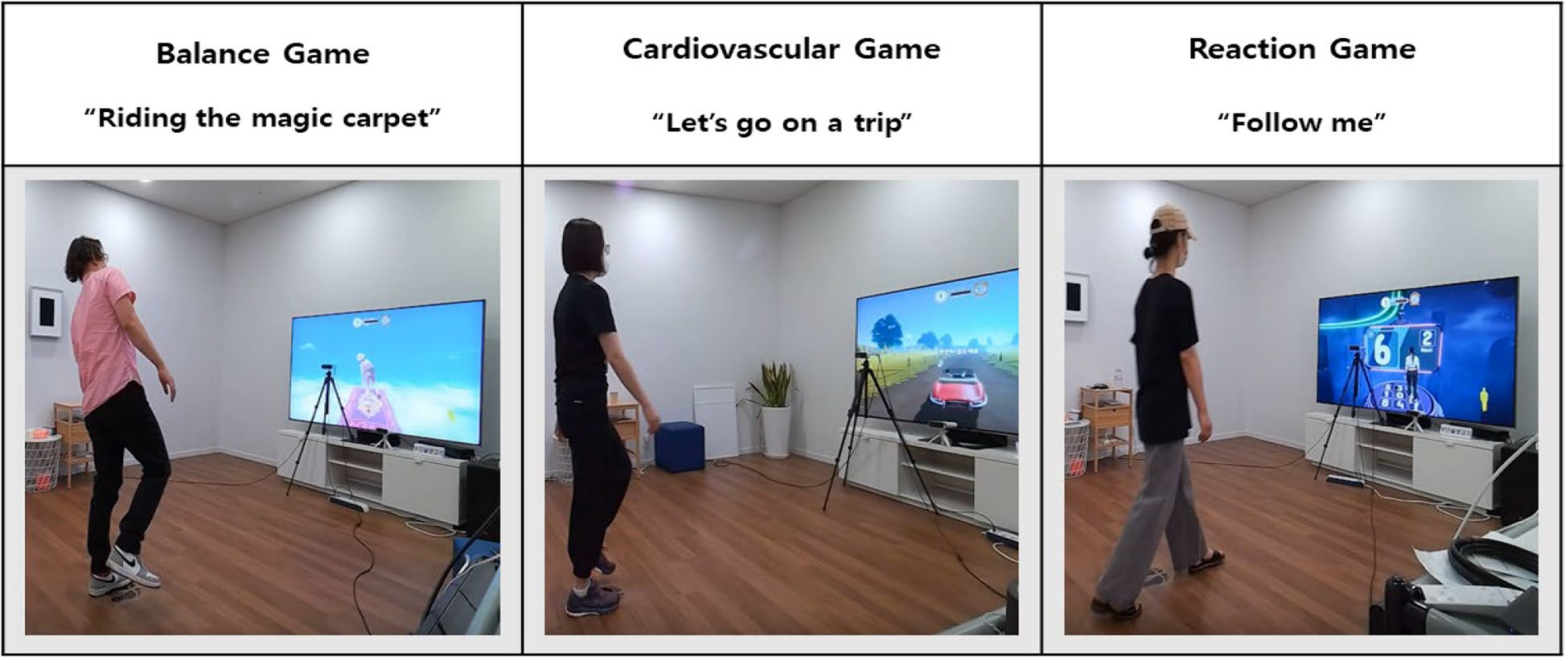
Example of participants performing the three exergames

Azure Kinect cameras were used to track body motion, allowing participants to control the game via physical body movement. Individuals with different movement skills, including a balance task, a cardiovascular fitness task, and a reaction task, were required to complete each of the games (Fig. 3). Before starting each game, a brief introduction to the game was displayed on the monitor.

Game 1, called *“Riding the magic carpet”*, consisted of a balance task that was used to evaluate participants’ postural sway. Within the game, participants were expected to control a mannequin flying on a magic carpet while sudden rushes of wind pushed the carpet into unstable positions. To prevent the mannequin from falling off the carpet, participants had to counteract the wind with body movement. The game was divided into 8 active phases requiring the participants to maintain balance and 8 resting phases of 5 s each in between. The difficulty of the game increased over time, with new wind rushes occurring every 4 s at the beginning and every 2 s at the end of the game.

For the cardiovascular game *“Let’s go on a trip”* (game 2), participants had to walk, jog, and run on the spot to move a simulated car, train, or bike along a racing track shown on the monitor. A brief introduction before starting the game was used to familiarize participants with the game and to prevent them from running across the room. The game was designed to challenge participants’ cardiovascular endurance, as they must maintain walking/jogging/running to prevent their vehicle from stopping. In total, the game lasted 5 min and was divided into 4 walking phases, 2 jogging phases and 2 running phases, with each phase lasting 30 s. Between each phase, a resting sequence of 10 s was inserted. The intensity of the game changed over time in an interval-style manner and was performed in the following order: walking (30 s), jogging (30 s), walking (30 s), running (30 s), walking (30 s), walking (30 s), jogging (30 s), and running (30 s). To control the intensity, acoustic signals were given throughout the game, with 70 bpm during walking, 156 bpm during jogging and 168 bpm during running.

**Fig. 4:**
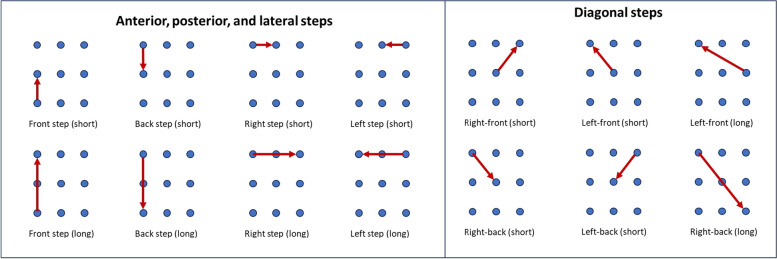
Fourteen step types included in game 3. The step types include movements in the anterior, posterior, lateral, and diagonal directions

In the last game, *“Follow me”*, participants had to move across a virtual field of 9 steps arranged in 3 rows and 3 columns according to different instructions presented on the screen in front of them. The instructions included different stimuli (with each stimulus representing one of the 9 steps) that participants had to respond to by moving their mannequin by stepping to the allocated step. The game included a total of 114 movements composed of 14 different step types, consisting of movements in the anterior, posterior, lateral and diagonal directions (Fig. 4).

**Fig. 5:**
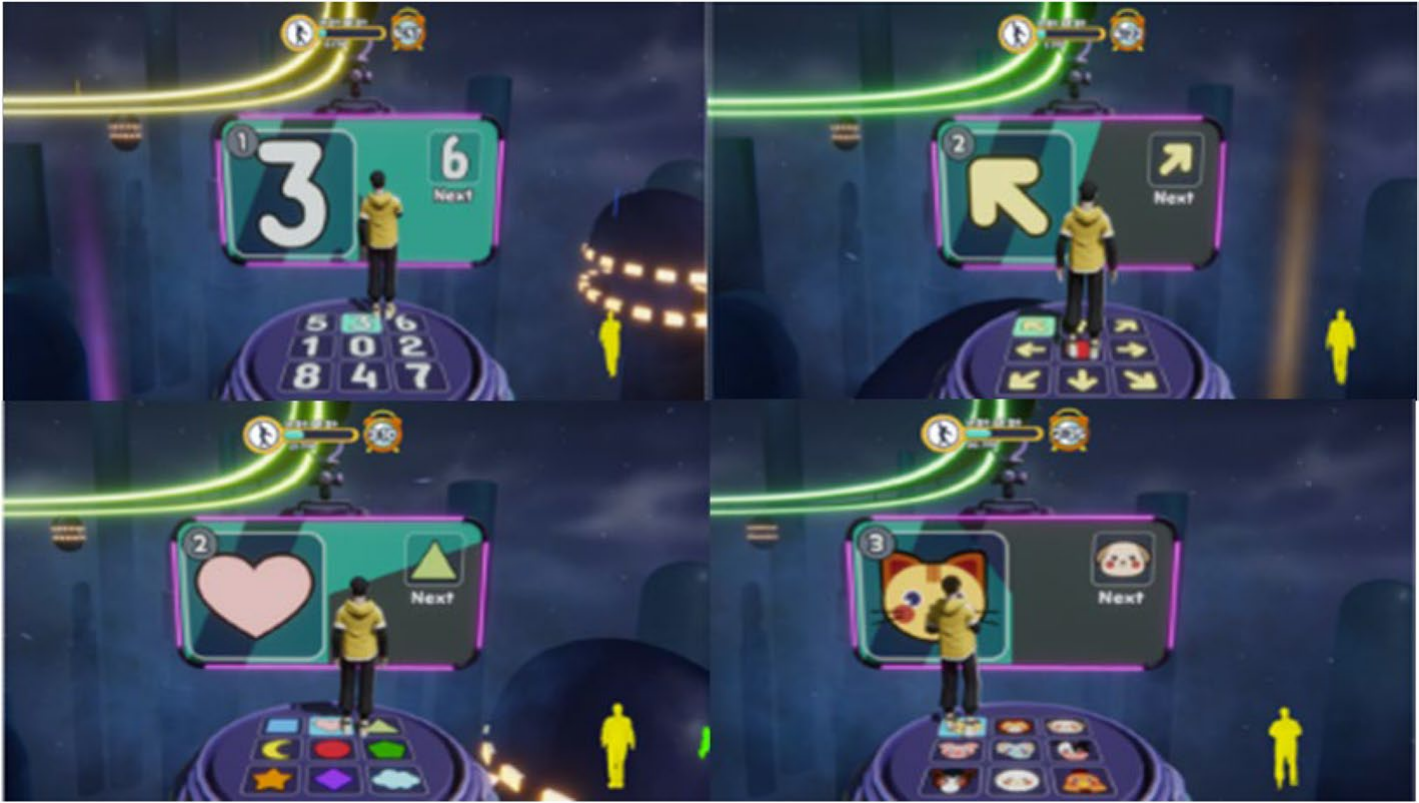
The stimuli are presented as numbers, arrows, shapes, and animals

While performing the game, both the stimulus representing the current movement and the stimulus for the following movement were presented at the same time (Fig. 5). Instructions were divided into 4 different blocks, with stimuli presented as numbers, arrows, shapes, and animals. Throughout the game, the blocks were repeated in the following order: numbers, arrows, numbers, arrows, shapes, animals, numbers, arrows, shapes, and animals.

The difficulty of the game varied over time and within the blocks, with a new stimulus being presented at a frequency of either every 4 s or every 2 s. Blocks including numbers and arrows were performed three times each, with stimuli presented at a frequency of 4 s in the first sequence and every 2 s in the second and third sequences. Blocks with shapes and animals were tested only twice, with stimuli changing after 4 s in the first trial and after 2 s in the second trial. If participants did not move their mannequin to the allocated step in the given time or moved it to a different step, the movement was considered a mistake.

### Intervention

**Fig. 6:**
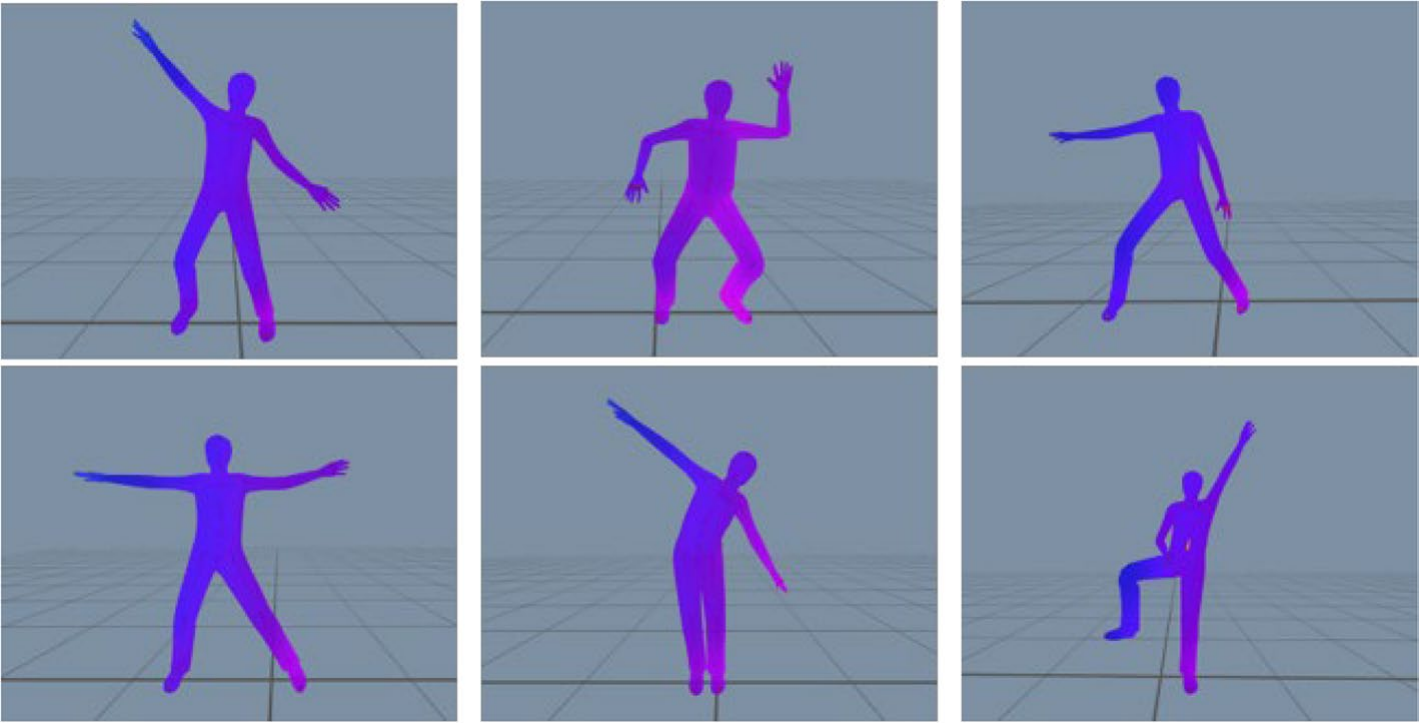
Screenshots of the exercise program showing an example movement sequence

In this study, a smartphone-based physical activity program developed by Hanyang University Digital Healthcare Center was used as an intervention over a period of 4 weeks. Depressed patients show decreased engagement in physical activity if exercise programs are too time intensive, too difficult to access, or require additional equipment [33]. To overcome these obstacles, the smartphone-based app was designed to provide a time-saving and easily accessible method of exercise that can be used anytime and anywhere without the need for additional exercise equipment. The physical activity promotion program implemented in the app consisted of a 20-minute-long YouTube video [34] aimed at promoting the users’ physical activity level and addressing health-related fitness components (flexibility, cardiovascular endurance, muscle strength). Exercises contained in the video included stretching drills to increase flexibility, continuous movements to stimulate the cardiovascular system, and yoga-based postures to hold for 10 to 30 s to strengthen muscles (Fig. 6) and followed the standard training procedure of 3 stages consisting of a warm-up, main exercise and cool down. The exercise group was instructed to use the program 5 times a week over a total period of 4 weeks. A duration of 4 weeks was chosen because it is the most commonly used duration of mobile health interventions for treating depressive disorders [35]. The control group was instructed not to change their usual exercise routine in daily life to increase or decrease their activity level and thereby influence outcome variables.

### Outcome measures

#### Centre for Epidemiological Studies Depression Scale (CES-D-10)

The CES-D-10 is a self-administered scale to assess symptoms of depression over the last week. It includes ten items (three on depressed affect, five on somatic symptoms and two on positive affect), rated from 0 (“rarely or none of the time”) to 3 (“nearly every day”). In summary, the total score can range from 0 to 30, with higher scores indicating greater symptom severity. The questionnaire is a reliable and valid tool for assessing depression symptoms and is quick and easy to use [36–38]. Cho and Kim [39] validated the Korean version of the Center for Epidemiologic Studies Depression Scale (CES-D). In the Korean version, one positively stated item from the original US English version was rephrased to convey a negative meaning. This was done to account for the reluctance of Asians to provide extremely positive responses to positively stated items. Additionally, the Korean version altered the scale using only “no” answers to a score of zero and “yes” to a score of one, following the shortened version known as the Boston form [40]. This leads to a total range from 0 to 10 in comparison to the international version, which ranges from zero to 30 [41]. The study revealed that the Korean version of the CES-D had good internal consistency, with Cronbach’s alpha coefficients of 0.91 in the general population and 0.89 in depressed patients. These results suggest that the Korean version of the CES-D is a reliable and valid tool for measuring depression in Korean populations [42].

#### Patient Health Questionnaire (PHQ9)

The PQH9 depression module (shortened form of the original PHQ assessment for mental disorders) is a self-administered questionnaire developed by Spitzer et al. [43] to assess symptoms and severity of depression. It contains nine items assessing the frequency of depressive symptoms over the last two weeks, each rated on a scale from 0 (“not at all”) to 3 (“almost every day”). The items are summed to a total score and are interpreted as follows: 0–4 points = “normal”, 5–9 points = “mild depression”, 10–14 points = “moderate depression”, 15–19 points = “moderately severe depression”, and 20 points or more “severe depression”. The PHQ9 module shows excellent reliability and high validity in diagnosing depression [44, 45].

#### YMCA step test

The cardiorespiratory capacity of each participant was measured based on heart rate recovery using the YMCA step test. Among the variety of submaximal step tests used to predict VO_2_max, the YMCA step test is commonly used [46]. Following the test protocol by Van Kieu et al. [46], the resting heart rate of each participant was first measured while they were sitting comfortably on a chair. Then, the participant had to step up and down a 30 cm box for 3 min following a metronome beat at 96 bpm (male high school students at 120 bpm). After 3 min, the participants were instructed to sit on a chair and rest for 1 min. After 1 min of rest, the heart rate was measured again, and VO_2_max was calculated using the following formula [46]:

$$\mathrm{Male}\;\mathrm{participants}:\;70.597\;-\;0.246\;\mathrm x\;\mathrm{age}\;+\;0.077\;\mathrm x\;\mathrm{height}\;-\;0.222\;\mathrm x\;\mathrm{weight}\;-\;0.147\;\mathrm x\;\mathrm{recovery}\;\mathrm{heart}\;\mathrm{rate}.\;\mathrm{Female}\;\mathrm{participants}:\;70.597\;-\;0.185\;\mathrm x\;\mathrm{age}\;+\;0.097\;\mathrm x\;\mathrm{height}\;-\;0.246\;\mathrm x\;\mathrm{weight}\;-\;0.122\;\mathrm x\;\mathrm{recovery}\;\mathrm{heart}\;\mathrm{rate}.$$

#### Grip strength

To evaluate grip strength, the maximum isometric strength of both the right and the left arms was measured using a Smedley hand grip dynamometer (Yagami Ltd., Tokyo, Japan). The participants were standing in an upright position, holding the handle of the dynamometer with the second knuckle of the fingers and the arm to be measured held at 15° and the elbow by the side of the body. The dynamometer was squeezed with maximum strength for a duration of 5 s. Relative grip strength was measured twice for both the right and the left arms, with the maximum value recorded in units of 0.1 kg. Research shows that grip strength can be influenced by individual differences among participants and recommends normalizing measured values by body weight [47, 48]. Therefore, grip strength values in this study were calculated as follows: grip strength (kg)/weight (kg)×100 (%).

#### Movement characteristics

While playing the games, body tracking was used to monitor and analyze the participants’ in-game kinematics in 3D using the Azure Kinect. A total of three Azure Kinect cameras were used to record movement at 15 Hz, and all the data were uploaded and saved on Microsoft’s Azure Cloud. The data were filtered using a 4th order Butterworth filter with a cutoff of 10 Hz before the calculation of kinematic variables and were stored in Microsoft Excel. Kinematic outcomes were calculated in MATLAB and included as follows: For the balance game, the total trunk movement (m) was calculated. During the cardiovascular game, the user’s body sway (mm), vertical head movement (mm), vertical hand swing (mm) and number of steps were calculated for walking, jogging, and running conditions, respectively. The outcomes analyzed for the reaction game included the user’s pelvis movement speed (m/s) for each of the four different stimulus types (numbers, arrows, shapes, animals) and the total number of mistakes made within 5 min.

During quantitative analysis of the three games, we found obvious differences in movement patterns and strategies to complete the game among participants within the reaction game (“Follow me”). Therefore, video data for this game were reviewed, and a qualitative analysis of the included motion was performed to describe these differences.

### Statistical analysis

All variables were analyzed using two-way (experimental versus control group) mixed model (pre and post-test)_ANOVA with a significance level of < 0.05 applied to investigate any significant differences. To estimate the effect of exercise on the significant variables, the effect size (ɳ^2^) was calculated.

## Results

### Participants

Prior recruitment for testing we used G Power (version 3.1.9.7) to calculate the total sample size of 24 for the number of participants based on and effect size of 0.3, alpha level of 0.05, power of 0.8, and 2 measurements. To try an account for the difficulties of unseen issues during the COVID period for the initial trial, a total of 68 participants were recruited by Hanyang University. After excluding participants with a CES-10-D score lower than three, 43 participants met the inclusion criteria for this study. Due to dropout during the intervention phase in eight patients and incorrect data in another four patients, 12 additional participants had to be excluded. This led to a total of 31 participants being included in the final data analysis, of which 21 participants were randomly assigned to the exercise group and 10 to the control group using Microsoft Excel random number generator. An approximate ratio of 2:1 of exercise group to control group was decided upon prior initiation of the intervention to increase the statistical power. Actual statistical power calculated by G Power was 0.74 based on the actual number of 21 participants, effect size of 0.3 with an alpha level of 0.05. Demographics for both the exercise and control groups are shown in Table 1.

**Table 1:** Participant demographics

	Exercise (*n* = 21)	Control (*n* = 10)
Age (years)	30.33 ± 8.97	26.00 ± 4.29
Height (m)	167.04 ± 8.88	168.84 ± 8.69
Weight (kg)	69.20 ± 26.04	65.28 ± 10.84
BMI	24.26 ± 6.45	22.78 ± 8.11
Gender	male:8	male:4
	female:13	female:6

### Depression scores

The results for the depression scores are reported in Table 2. Mixed model ANOVA was used to examine the effects of group (exercise vs. control) and measurement (pre vs. post) on depression scores. Box’s test of equality of covariance shows no significant differences covariance within each variable. There was a significant main effect of measurement for both the CES-10-D-Score (*F*_*1,29*_ = 89.99, *P* < .001; η²=0.76) and the PHQ9-Score (*F*_*1,29*_ = 13.68, *P* = .001; η²= 0.32), indicating reduced depression symptoms after 4 weeks compared to baseline for both groups. There was no significant group effect or group measurement interaction.

**Table 2:** Participant depression scores according to group and testing time

	Exercise Group		Control Group		*Mixed Model ANOVA*		
	Pre	Post	Pre	Post	Group x Time (*P* value)	Group (*P* value)	Time (*P* value)
CES-10-D	6.43 ± 2.56	2.86 ± 2.39	6.40 ± 2.59	2.40 ± 2.67	0.59	0.79	< 0.001
PHQ9	9.19 ± 4.58	6.33 ± 4.10	10.90 ± 6.33	6.40 ± 3.75	0.42	0.55	0.001

### Fitness scores

In the YMCA step test, both the exercise and control groups had lower postintervention heart rates than did the baseline group, even though there were no significant main effects for measurement (*F*_*1,29*_ = 1.03, *P* = .32; η²= 0.03), group (*F*_*1,29*_ = 0.07, *P* = .79; η²= 0.00) or group interaction (*F*_*1,29*_ = 0.00, *P* = .98; η²= 0.00). A reduced heart rate induces an improvement in cardiovascular fitness.

While grip strength increased in the exercise group for the left and right hands, the control group showed a reduction in strength in both hands. These differences were not statistically significant. The left hand showed no effects for measurement (*F*_*1,29*_ = 0.03, *P* = .86; η²= 0.00), group (*F*_*1,29*_ = 0.14, *P* = .71; η²= 0.01), or group measurement interaction (*F*_*1,29*_ = 3.95, *P* = .06; η²= 0.12). Similarly, there was no main effect for the right-hand regarding measurement (*F*_*1,29*_ = 0.03, *P* = .87; η²= 0.00), group (*F*_*1,29*_ = 0.25, *P* = .62; η²= 0.01), or group measurement interaction (*F*_*1,29*_ = 1.87, *P* = .18; η²= 0.06). All the results are listed in Table 3.

**Table 3:** Participant fitness scores according to group and testing time

		Exercise Group		Control Group		*Mixed Model ANOVA*		
		Pre	Post	Pre	Post	Group x Time (*P* value)	Group (*P* value)	Time (*P* value)
VO_2_max		102.24 ± 20.61	99.52 ± 26.41	104.50 ± 25.15	101.90 ± 19.58	0.98	0.79	0.32
Grip strength (%)	left	28.51 ± 9.01	29.50 ± 9.09	28.16 ± 8.15	27.33 ± 8.49	0.06	0.71	0.86
	right	30.55 ± 9.59	31.58 ± 8.68	29.77 ± 8.28	28.99 ± 8.16	0.18	0.62	0.87

### Movement characteristics

All results for the in-game kinematics are presented in Table 4.

#### Cardiovascular game

For the exercise group, the number of steps decreased across all conditions (walking, jogging, running), while the number of steps increased in the control group. In the walking condition, there was a significant main effect of group (*F*_*1,29*_ = 6.93, *P* = .01; η²= 0.19), with the control group doing more steps than the exercise group. For measurement (*F*_*1,29*_ = 0.04, *P* = .84; η²: 0.00) and group measurement interaction (*F*_*1,29*_ = 2.74, *P* = .11; η²= 0.09), no significant differences were found. For the jogging and running conditions, no significant differences were found between the groups or between the measurements.

The vertical hand swing showed a decrease across all the conditions for both the exercise and control groups, although none of the differences were significant for either the measurement, group or group measurement interaction.

While the exercise group showed increased total body sway post measurement for all three conditions, body sway decreased in the control group. In the jogging condition, a significant main effect for group measurement interaction was observed (*F*_*1,29*_ = 4.38, *P* = .045; η² = 0.13). Similarly, head movement increased in all conditions in the exercise group but decreased in the control group. Again, the jogging condition had a significant main effect on the group measurement interaction (*F*_*1,29*_ = 6.30, *P* = .02; η² = 0.18).

#### Balance game

For the balance game, the total movement of the trunk was measured. Both groups showed increased trunk movement, but no significant main effects were found for measurement (*F*_*1,29*_ = 1.66, *P* = .21; η² = 0.05), group (*F*_*1,29*_ = 2.70, *P* = .11; η² = 0.09) or group measurement interaction (*F*_*1,29*_ = 0.01, *P* = .91; η² = 0.00).

#### Reaction game

By analyzing the reaction game, the number of mistakes and the movement speed of the trunk when reacting to different stimuli (animals, arrows, numbers, shapes) were calculated. Regarding the number of mistakes, there was a significant interaction effect of group measurement (*F*_*1,29*_ = 5.53, *P* = .03; η² = 0.16), with a decrease in the exercise group and an increase in the number of mistakes in the control group. No significant interaction effects were found for movement speed among the four different types of stimuli.

**Table 4:** Participant in-game kinematics for the 3 games

	Exercise Group		Control Group		*Mixed Model ANOVA*		
	Pre	Post	Pre	Post	Group x Time (*P* value)	Group (*P* value)	Time (*P* value)
**Cardiovascular Game**							
No. of steps – walk (No.)	372.62 ± 35.60	357.52 ± 49.84	394.30 ± 27.37	406.00 ± 39.01	0.11	0.01	0.84
No. of steps – jog (No.)	195.76 ± 17.92	185.48 ± 24.04	197.60 ± 25.69	191.70 ± 17.28	0.64	0.56	0.09
No. of steps – run (No.)	288.29 ± 26.47	287.00 ± 31.53	302.50 ± 35.87	278.80 ± 43.44	0.14	0.77	0.09
Hand swing – walk (mm)	206.19 ± 119.73	174.86 ± 113.57	248.73 ± 114.03	195.16 ± 78.22	0.63	0.39	0.07
Hand swing – jog (mm)	120.79 ± 116.04	112.24 ± 75.09	107.74 ± 59.68	89.72 ± 34.05	0.72	0.56	0.32
Hand swing – run (mm)	123.49 ± 93.05	104.98 ± 61.06	111.98 ± 75.06	82.59 ± 38.88	0.70	0.49	0.09
Body sway - walk (mm)	35.82 ± 20.41	42.55 ± 18.67	38.78 ± 6.98	33.56 ± 17.19	0.08	0.62	0.82
Body sway –jog (mm)	23.83 ± 13.68	29.21 ± 10.33	27.99 ± 4.92	24.34 ± 10.44	0.045	0.92	0.69
Body sway – run (mm)	20.81 ± 12.10	24.61 ± 9.14	23.80 ± 3.96	23.27 ± 8.57	0.28	0.79	0.41
Head movement-walk (mm)	44.37 ± 29.40	53.72 ± 27.73	51.96 ± 13.15	45.16 ± 23.67	0.09	0.96	0.78
Head movement – jog (mm)	32.68 ± 20.50	41.58 ± 19.59	38.70 ± 9.42	33.38 ± 13.88	0.02	0.86	0.53
Head movement –run (mm)	34.69 ± 21.12	38.49 ± 16.09	37.13 ± 8.66	35.45 ± 13.84	0.32	0.96	0.69
**Balance Game**							
Total trunk movement (m)	16.22 ± 4.71	17.36 ± 3.30	18.38 ± 2.73	19.33 ± 4.06	0.91	0.01	0.21
**Reaction Time Game**							
No. of mistakes	0.71 ± 0.90	0.52 ± 81	0.10 ± 0.32	0.90 ± 1.66	0.03	0.70	0.16
Trunk speed – Animals Icons (mm/s)	390.22 ± 24.72	379.29 ± 30.94	382.63 ± 47.34	392.33 ± 31.11	0.28	0.17	0.94
Trunk speed – Arrows Icons (mm/s)	408.52 ± 30.69	396.84 ± 24.81	403.34 ± 30.88	411.32 ± 35.15	0.78	0.95	0.93
Trunk speed – Numbers Icons (mm/s)	410.71 ± 89.25	407.35 ± 43.72	406.96 ± 48.90	413.17 ± 33.76	0.26	0.54	0.83
Trunk speed – Shapes Icons (mm/s)	381.56 ± 40.93	368.85 ± 42.53	386.87 ± 54.05	397.83 ± 30.83	0.14	0.79	0.93

### Qualitative findings

Qualitative analysis of the video data revealed interindividual differences in movement patterns and game strategies among participants. Depending on the depression score, differences in step patterns (step length, number of steps) and in the timing of responding to the stimuli were observed: more depressed participants tended to take numerous small steps to reach the intended step, while less depressed participants tended to take only one step with an increased step length. In addition, there were differences in performing the step, with some participants using both legs to move toward the intended field, while others stayed in a central position with one leg and only moved to the intended step with the other leg. Another observation was a difference in response timing to the presented stimuli. While less depressed participants waited for the current stimulus to finish and then moved according to the following stimulus, participants with higher depression scores tended to not wait for the current stimulus to finish and moved according to the following stimulus immediately.

## Discussion

The results of this study showed no significant intervention effects of a 4-week mobile phone-based physical activity program on depression scores (CES-D-10 and PHQ-9) or fitness levels (YMCA step test and grip strength) compared to the control condition. Although both depression scores decreased significantly after 4 weeks, the same effect was observed for the controls who did not use the program. However, this study showed that mobile phone-based activity affected participants’ movement characteristics within the Azure Kinect exergames. In the cardiovascular fitness game jogging condition, we found an increase in body sway and vertical head movement in the exercise group but a decrease in the control group. For the walking condition, a significant group effect was observed for the number of steps, with steps increasing in the control group and decreasing in the exercise group. In addition, a main interaction effect was found regarding the number of mistakes made in the reaction game, with a decrease in the exercise group and an increase in the number of mistakes in the control group. Qualitative analysis of the reaction game showed differences in step patterns and in temporal reaction to a presented stimulus among participants.

To our knowledge, this is the first study to perform a comprehensive analysis of differences in the movement characteristics of depressed participants following a mobile phone-based physical activity intervention. Existing research has focused mainly on differences between depressed patients and healthy controls but has not included changes in movement following a physical intervention. In a 2-month walking intervention study of depressed adults, Deschamps et al. [49] reported improvements in postural control while walking, but the effects on other movement characteristics were not investigated. Therefore, the novel findings of this study contribute to the understanding of the relationship between depressive disorders and human movement.

### Depression score

Contrary to current research [26, 50, 51], the intervention in this study did not result in a significant interaction effect on depression symptoms. Although the depression scores (CES-D-10 and PHQ-9) significantly decreased in the exercise group, the same reduction was observed in the control group, which did not use the activity promotion program. For both groups, a significant reduction in the CES-D-10 score represented a change from a moderately depressed score to a mildly depressed score [52]. The PHQ-9 score also decreased significantly in both groups but still represented the same category of mild depression [44] as in the pretest. The results for the exercise group were comparable to those of a 4-week physical intervention study [53], which reported a reduction in the Hospital Anxiety and Depression Scale score from 6.0 ± 3.9 to 4.1 ± 3.3, representing similar score changes within the mild classification [54]. Other research [55] shows the significant improvements in depression score can be effected if the participant is provided a placebo or by participating in a study [56]. Interestingly, in a meta-analysis study focusing on the effect of placebo the authors recommending clinicians to use the power of the placebo and that researchers should try to control for its effects [57]. According to Hu et al. [58], these scores indicate that the user should be monitored and participate in a healthy lifestyle, such as regular exercise, to prevent depression.

There are various mechanisms, such as reduced inflammation, stress reduction, improved sleep, social interaction [59] that have been reported about the effect that regular exercise has on depression related behaviors, as well as the increase in brain-derived neurotrophic factor, and serum testosterone [60]. In other research, authors state that endorphins may provide the key to the effect of regular exercise on physiological factors that regulate psychological conditions [61]. Taheri and colleagues [62] highlights that even if elite athletes reduce their physical activity their stress and anxiety levels increase, which the authors believe is due to the ability of exercise to modulate the hippocampus plasticity therefore alleviating cognitive malfunction in depressed individuals.

A possible reason for the significant decrease in depressive symptoms in the control group might be the change in season occurring during the time of data collection. In a cross-sectional study, Lukmanji et al. [63] showed seasonal variation in reported depression symptoms, with an increase during the winter month compared to the summer month. The tests in our study were performed between April and July, when the weather became warmer and hours of sunshine increased in South Korea. These climatic changes might have influenced participants’ moods and their reporting of depression levels. Additionally, social distancing rules due to the COVID-19 pandemic were loosened in Seoul, South Korea, from the beginning of April [64]. Restrictions on private gatherings were reduced, and operating times for daily living facilities were extended. This might have had a major influence on the mood of the participants in this study and might have led to a bias in the reported depression score. It should also be noted that the exercise group benefited from loosened restrictions in the same way as the control group. Therefore, the influence of physical intervention on depressive symptoms in this study remains unclear, and further research with fewer confounding factors is needed to investigate the effectiveness of mobile phone-based physical treatment.

### Differences in fitness

Mental health is related to regular physical activity, with people exercising more frequently showing lower odds of developing depressive symptoms. Additionally, the odds of frequent depression symptoms are reduced in a dose‒response manner with higher cardiovascular fitness levels [65]. In this study, participants in both the exercise and control groups showed no significant reduction in heart rate after completing the YMCA step test. Likewise, we found no significant changes in grip strength in the exercise group compared to the control group. In a review on the effects of exercise training on handgrip strength, Labott et al. [66] showed that there is only a small effect of physical interventions on the increase in grip strength. Although not significant, the exercise group showed descriptive increases in grip strength in both the right and the left hands, while the control group performed worse in the posttest than in the pretest. This might be an indication for possible changes in grip strength if the exercise time was increased, providing more time for grip strength to increase, as reported in rehabilitation settings that apply gamification for increased engagement and adherence to treatments [67]. Like cardiovascular fitness, grip strength is a health indicator inversely associated with an increased risk of death, poorer mental health, and increased odds of depression [68, 69]. This relationship might explain the reduction in the depression score in the exercise group, while grip strength simultaneously increased.

### Movement characteristics

The second objective of this study was to investigate the effect of the intervention on in-game movement characteristics. Depression disorders are associated with alterations in locomotion and thereby influence movement [70]. When comparing the movement characteristics of depressed and nondepressed participants, prior research has focused primarily on analyzing differences in gait patterns, showing that depressed patients are characterized by reduced gait velocity, vertical movement, and stride length, as well as increased gait cycle duration [29–31, 71].

In this study, we likewise found a significant increase in vertical head movement in the exercise group for the cardiovascular game jogging task. In contrast, the control group showed a slight decrease in vertical movement. When Adolph et al. [29] compared gait patterns between patients with major depressive disorder and a matched nondepressed control group, they found that depressed patients showed reduced vertical up-and-down movements. A similar relationship between depression disorder and decreased amplitudes of vertical movements was found by Michalak et al. [30] via laboratory gait analysis. According to these results, the changes in movement characteristics in the exercise group might be an indicator of a reduction in depression. On the other hand, our findings of increased body sway in the exercise group are contrary to prior research showing increased body sway in gait for depressed patients [30, 31]. Differences might occur due to different movement requirements in the exergame of this study, e.g., walking on the spot, compared to walking along a straight line, but further investigation on body sway in-game situations is needed to compare common alterations in gait patterns. The movement pattern changes (body sway and head movement) shown in this study in gait and jogging may be clinically significant from the aspect of the strong link between gait characteristics and participants with depression [72].

In addition to alterations in movement characteristics, previous research has shown that cognitive dysfunction is one of the main criteria for depressive disorders [73, 74]. In the present study, there was a main effect for mistakes made in the reaction game, with participants in the exercise group improving their performance by making fewer mistakes after the 4-week intervention. According to Keller et al. [73], depressed individuals perform worse in tasks that include selective attention than healthy controls. Impairments in attentional focus within higher depression levels might be a possible explanation for the differences between pre- and postintervention testing within the exercise group and might indicate an improvement in attentional focus in the exercise group following physical intervention. In contrast to the expected learning effect, the number of mistakes increased in the control group post-test and decreased in the exercise group, even though both groups showed decreases in depression scores after 4 weeks. These notable results linking the correlation between attentional focus, physical activity and depression level require further investigation to provide more meaningful explanations of their relationship.

During the analysis of the video data, we found that independent of group affiliation, participants showed large interindividual differences in movement patterns and game strategies within the reaction game. These differences occurred due to instructions within the games being kept to a minimum to not limit participants’ particular movement patterns. We observed that some participants moved only one leg to follow the stimuli while remaining centered with the other leg, whereas other participants followed using both legs. These interindividual variations resulted in large movement differences between participants, making it difficult to perform meaningful quantitative analysis of parameters such as step length. Therefore, the videos were also checked for qualitatively visible differences among the participants. In doing so, we found that participants with lower depression scores tended to step longer than those with higher depression scores. These findings are consistent with prior research showing that depressed patients differ in gait with a shorter stride length [29, 30]. Qualitative analysis also revealed differences in the reactions to the presented stimuli. While not only the current presented stimuli but also the following stimuli were visible to the participants, participants with higher depression scores tended to react to the following stimuli before they were initially meant to do so. On the other hand, less depressed participants used to wait for the switch between the current and following stimuli before reacting. To expand the analysis of in-game movement patterns, further research should also consider including stricter limitations of possible movements to make them quantitatively comparable.

### Limitations and future research

A limitation of this study is the small sample size, leading to decreased statistical power that might have influenced the observed results. In addition, the convenience sampling method used in this study might have led to sampling bias in the results. The data analyzed to compare movement characteristics were extracted from an initial clinical trial investigating differences in behavioral characteristics between depressed patients and nondepressed controls. Unfortunately, not all of the 68 participants included in the initial trial were instructed to perform all three Azure Kinect games. In accordance with the literature, we decided that all the motor skills included in the three games were essential to address our study objectives and therefore had to exclude multiple participants, which also led to an uneven distribution of participants between the two groups. To avoid bias, future research should therefore increase the statistical power by including more participants, balancing treatment groups, and control groups equally. Additionally, future research should include some of the other factors that might affect depression and the effectiveness of physical activity, such as sleep characteristics and overall lifestyle [1].

Another limitation was that additional activity levels of both groups before and during the intervention were not monitored or recorded. Exercising 20 min a day for 5 days a week might have been a significant increase in time being active for some of the participants, while it might have been no increase for others at all. Although the participants in the exercise group reported having used the game on a regular basis, the exact duration of weekly gameplay was not measured, making it difficult to control the actual effect of the activity promotion program on participants’ exercise behavior. The program included only a single mobile phone-based workout and might have become boring over the 4 weeks, leading to less active participation in the program. In addition, the amount of physical activity and other factors that affect depression, such as lifestyle, i.e., sleep characteristics, should be measured using smart watches to be included in future research. This will help to assess the amount of activity the program adds to participants’ usual exercise routine and therefore make it easier to draw conclusions on the effect of the program.

In addition, there were many external confounding factors that might have had an essential influence on the outcomes of this study. As described earlier, restrictions on social distancing were loosened, and the climatic season changed during the time participants underwent the intervention. We assume that this had a substantial impact on participants’ depression scores, leading to a decrease in depression levels in both groups. It is possible that the observed changes in depression symptoms in either of the groups did not occur without the effects of these confounding factors.

Despite these limitations, the results of this study add new knowledge to the understanding of movement characteristics in depressed patients. We showed that vertical head movement while jogging, as well as the number of mistakes in a reaction task, was positively affected by the intervention. These findings support the idea that activity-promoting mobile games are a promising option for the detection and treatment of depressive disorders. Nevertheless, more research is needed to validate the findings of this study. The qualitative differences in step patterns and temporal reactions to a presented stimulus found between participants should be included in further investigations. Additionally, future research might consider a prolonged intervention time and longer exercise times for more substantial effects due to an apparent dose‒response relationship between exercise and mental health [75].

## Data Availability

Data and materials are not publicly available due to privacy concerns. The data that support the findings of this study may be available from Hanyang University Digital Healthcare Center, but restrictions apply to the availability of these data, which were used under license for the current study, and so are not publicly available. In general data and associated materials are not publicly available due to privacy concerns. For access to the data please contact the Hyungsook Kim at Hanyang University by email at khsook12@hanyang.ac.kr.
